# Analog neuromorphic circuit for spontaneous Ca^2+^ oscillations

**DOI:** 10.1038/s41598-023-47433-w

**Published:** 2023-11-16

**Authors:** Beatriz O. Câmara, Janaina G. Guimarães, Marcelo L. Pereira Junior

**Affiliations:** 1 Department of Electrical Engineering, Faculty of Technology, University of Brasília, 70910-900 Brasília, Brazil; 2 Federal University of Santa Catarina, Control Engineering, Automation and Computer Science, 89036-256 Blumenau, Santa Catarina Brazil

**Keywords:** Biotechnology, Neuroscience, Neurology, Engineering, Mathematics and computing

## Abstract

This study proposes an innovative analog neuromorphic circuit design to mimic spontaneous Ca^2+^ oscillations observed in astrocytes. Unlike traditional models, this approach does not rely on synaptic stimulation, suggesting that astrocytes may play a key role in generating neuronal activity. The circuit is built using transistor differential pairs to approximate the nonlinear sigmoidal biological functions, and its performance is validated through simulation and compared against mathematical models using phase diagram analysis. Results indicate a good fit between the circuit and the mathematical model. Finally, the circuit’s ability to simulate the release of glutamate and ATP through spontaneous oscillations is demonstrated.

## Introduction

Artificial neural networks (ANNs) have become an industry of great impact on society, with continuous growth and improved state-of-the-art achieved through the development of new algorithms and better learning techniques^1^. However, despite the current impressive applications of ANNs, there is still much to be explored in the field of cognitive computing, as the mechanisms behind intelligence and the brain itself remain largely unknown. Neuroscientists are constantly making new discoveries that have the potential to improve the implementation of ANNs, and vice versa, as engineering feedback sheds light on biological questions^2–4^. As such, the interdisciplinary relationship between neuroscience and engineering continues to be of utmost importance for advancing the field of artificial intelligence.

Astrocytes, abundant glial cells in the brain, have emerged as a subject of interest in recent researches. Over the last few decades, studies have revealed that astrocytes play a crucial role in neural network communication and modulation^5–7^. They form networks that are believed to be as important as the neural networks themselves. Research also suggests that astrocytes are involved in memory formation and possibly neurodegenerative disorders^7–9^.

The encoding and transfer of information between astrocytes and other cells are achieved through intra and intercellular Ca^2+^ oscillations, which are modulated by synaptic activity and, in turn, regulate these activities by releasing gliotransmitters such as glutamate, ATP, or adenosine in the synaptic cleft^10–14^.There are two types of Ca^2+^ oscillations: neurotransmitter-evoked and spontaneous^14^.

Neurotransmitter-evoked Ca^2+^ oscillations refer to oscillations that are generated as a response to synaptic activity^14^. During the synaptic transmission, the presynaptic neuron releases neurotransmitters that act upon both the postsynaptic neuron and the astrocyte. The result of this interaction is the generation of Ca^2+^ oscillations in the astrocyte^6,15^. The majority of the work about neuro-glia interaction refer to this type of oscillations, including mathematical models, and digital and analog neuromorphic implementations.

The main mathematical models for neurotransmitter-evoked Ca^2+^ include the Li-Rinzel model^16^, the Postnov model^10^ and the De Pittà model^11^. These models have been previously implemented as neuromorphic circuits. Nazari et al.^17^, Gomar et al.^18^, Hayati et al.^19^, Faramarzi et al.^20^, and Haghiri et al.^21^ are some examples of a digital implementation of these models. Analog neuromorphic implementations examples include Ahmadi et al.^22^, Ranjbar et al.^23^, and Khosravi et al.^24^.

Unlike the neurotransmitter-evoked oscillations, the spontaneous Ca^2+^ oscillations occur in the absence of external stimuli, being initiated by small variations in the cytosolic Ca^2+^ concentration ([Ca^2+^]_cyt_)^25–27^. Spontaneous Ca^2+^ oscillations are a common phenomenon observed in various cell types, including pituitary cells^28^ and cardiomyocytes^29^. Notably, such type of oscillations have also been detected in the cytosol of astrocytes^25–27,30^.

Recent studies have suggested that spontaneous oscillations in astrocytes may play an important role in astro-neural networks by initiating the process that leads to neural signaling^26,30^. However, the current mainstream paradigm in artificial neural network (ANN) applications assumes that astro-neural communication always starts with the neuron. This assumption provides an opportunity for further exploration of the potential contributions of astrocytes to the generation of neuronal signaling.

Some research in this area includes mathematical models for spontaneous oscillations^26,27,31,32^ and digital neuromorphic circuit implementations^33,34^. However, there is currently no proposal for an analog neuromorphic circuit capable of generating spontaneous Ca^2+^ oscillations.

Information is represented by relative values of analog signals in biological systems, making analog circuits a logical approach for the implementation of ANNs. It is possible to design very efficient neuromorphic circuits by defining an adequate representation of the biological variables, such that the physical principles and basic operations of analog devices match those of their biological counterparts. The advantages of an analog neuromorphic implementation include, but are not limited to, large-scale adaptivity, robustness to system failure and degradation, and low power consumption^35^ when compared to digital or non neuromorphical implementations.

This paper presents an analog neuromorphic circuit implementation of spontaneous Ca^2+^ oscillations based on the Lavrentovich-Hemkin (LH) model^26^. The LH model takes into account the mechanisms of inositol cross-coupling (ICC) and calcium-induced calcium release (CICR), as well as the independent production and degradation of inositol (1,4,5)-triphosphate (IP_3_). It assumes that a small change in cytosolic Ca^2+^ concentration due to the influx of calcium ions through the astrocyte’s membrane acts as the trigger for spontaneous oscillations.

While the LH model^26^ has previously been implemented as digital circuits^33,34^, this work proposes an analog neuromorphic system that aims to take advantage of the physical principles and VLSI properties of transistor-based circuits. Section “Spontaneous Ca^2+^ oscillations model” describes the LH mathematical model, while Section “Proposed circuit” presents the design and implementation of the model as circuits. The simulation and analysis of the circuits are discussed in Section “Results”, and the paper concludes in Section “Conclusion”.

## Methods

### Spontaneous Ca$^{2+}$ oscillations model

**Figure 1:**
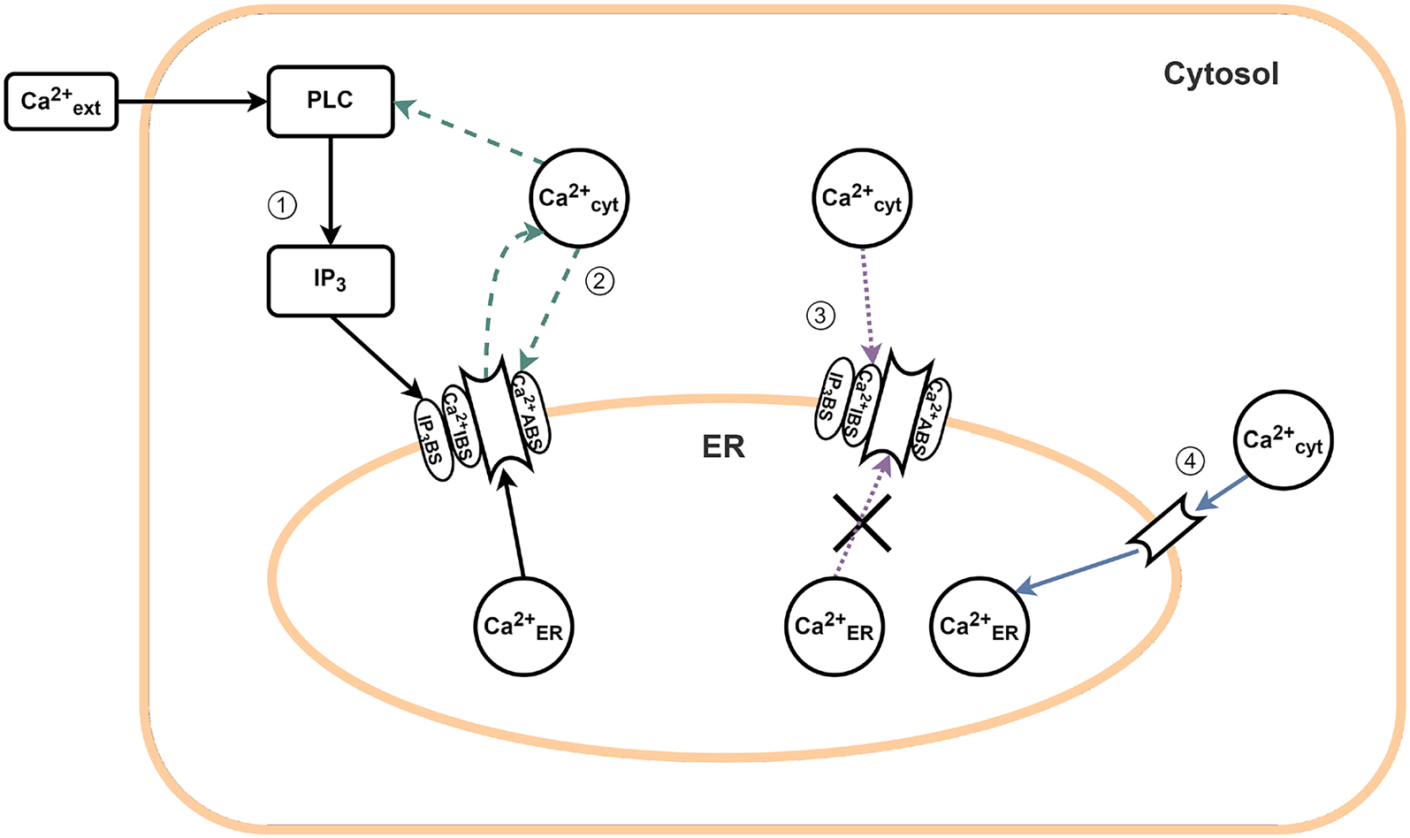
Schematic illustration of the mathematical model, showing paths 1 and 2 for the positive feedback loop and paths 3 and 4 for the negative feedback loop.

The Lavrentovich-Hemkin model^26^ describes the dynamic interactions of three variables: cytosolic Ca^2+^ concentration ([Ca^2+^]_cyt_), endoplasmic reticulum Ca^2+^ concentration ([Ca^2+^]_ER_), and cytosolic IP_3_ concentration ([IP_3_]_cyt_). The model considers the endoplasmic reticulum (ER) as the only Ca^2+^ storage unit and assumes the presence of a single type of receptor channel protein, the IP_3_ receptor (IP_3_R), with three binding sites: one for IP_3_, one for activating Ca^2+^, and one for deactivating Ca^2+^ (see Fig. 1).

The oscillation process is initiated by a small change in the concentration of Ca^2+^ in the cytosol ([Ca^2+^]_cyt_), which is a result of the influx of calcium ions through the astrocyte’s membrane from the extracellular space. The change in [Ca^2+^]_cyt_ levels triggers the production of inositol 1,4,5-trisphosphate (IP_3_) by the enzyme phospholipase C$\delta$1 (PLC$\delta$1). The IP_3_ then binds to the IP_3_ receptor (IP_3_R) on the endoplasmic reticulum (ER) membrane, causing the channel to open and allowing the flow of calcium ions from the ER into the astrocyte’s cytosol (path 1 in Fig. 1).

The channel opening is also regulated by the binding of Ca^2+^ to the activating binding site on IP_3_R, through a process known as calcium-induced calcium release (CICR) mechanism (depicted as path 2 in Fig. 1). The channel opening by the binding of both IP_3_ and Ca^2+^ initiates a positive feedback loop, leading to an increase in the concentration of Ca^2+^ in the cytosol.

Low concentrations of Ca^2+^ in the cytosol promote the activation of the IP_3_ receptor’s binding site. However, high concentrations of calcium ions inhibit the IP_3_ receptors by binding to the deactivating site, which results in the closure of the channel and the cessation of the Ca^2+^ flux from the ER into the cytosol (illustrated as path 3 in Fig. 1).

During this process, calcium ions are sequestered back into the ER through a mechanism known as the sarco(endo)plasmic calcium ATPase (SERCA) pump, thus decreasing the cytosolic calcium concentration (path 4 in Fig. 1). Both the Ca^2+^ deactivating binding and SERCA pumps generate a negative feedback loop. The interaction of the coupled positive and negative feedback loops perpetuates the Ca^2+^ oscillation mechanism. The system dynamics are described by three ordinary differential equations (1–3), where $X=[Ca^{2+}]_{cyt}$, $Y=[Ca^{2+}]_{ER}$, and $Z=[IP_3]_{cyt}$.

1 $$\begin{aligned}{} & {} \frac{dX}{dt} = V_{in}-k_{out}X+V_{CICR}-V_{SERCA} +k_f(Y-X) \end{aligned}$$

2 $$\begin{aligned}{} & {} \frac{dY}{dt} =V_{SERCA}-V_{CICR}-k_f(Y-X) \end{aligned}$$

3 $$\begin{aligned}{} & {} \frac{dZ}{dt} =V_{PLC}-k_{deg}Z \end{aligned}$$

The three main nonlinear terms in Eqs. (1–3) are described as follows:

- *V*_SERCA_ represents the Ca^2+^ flux from the cytosol to the ER due to the SERCA pumps (Eq. 4).
- *V*_PLC_ describes the formation of IP_3_ as a result of PLC$\delta$1 stimulation by calcium ions (Eq. 5).
- *V*_CICR_ describes the Ca^2+^ flow from the ER to the cytosol mediated by the IP_3_R (Eq. 6).

4 $$\begin{aligned}{} & {} V_{SERCA} =v_{M2}\left( \frac{X^2}{X^2+k_2^2}\right) \end{aligned}$$

5 $$\begin{aligned}{} & {} V_{PLC} =v_p\left( \frac{X^2}{X^2+k_p^2}\right) \end{aligned}$$

6 $$\begin{aligned}{} & {} V_{CICR} =\, 4v_{M3} \left( \frac{k_{CaA}^nX^n}{(X^n+k_{CaA}^n)(X^n+k_{CaI}^n)}\right) \times \left( \frac{Z^m}{Z^m+k_{ip3}^m}\right) (Y-X) \end{aligned}$$

The parameters’ values for these six equations can be obtained from the original work^26^ and are listed in Table 1. They are briefly explained below:

- *V*_in_—Ca^2+^ flux from the extracellular space into the astrocyte;
- *v*_M2_—maximum flux of Ca^2+^ through the SERCA pumps;
- *v*_M3_—maximum flux of Ca^2+^ into the cytosol from the ER;
- *v*_p_—maximum rate of IP_3_ formation;
- *k*_2_—Ca^2+^ concentration that achieves 50% of *v*_M2_’s maximum velocity;
- *k*_CaA_—IP_3_R activating affinity with Ca^2+^;
- *k*_CaI_—IP_3_R inhibiting affinity with Ca^2+^;
- *k*_ip3_—IP_3_ concentration that gives rise to 50% of *v*_M3_’s maximum velocity;
- *k*_p_—Ca^2+^ concentration that gives rise to 50% of *v*_p_’s maximum velocity;
- *k*_deg_*Z*—rate of degradation of IP_3_;
- *k*_out_*X*—rate of calcium efflux from the cytosol to the extracellular space;
- *k*_f_*(Y-X)*—leak flux from the ER into the cytosol;
- *n* and *m*—Hill coefficients.

**Table 1:** Parameter’s values for the spontaneous Ca^2+^ oscillations model^26^.

Parameter	Value
V_in_	0.05 μM/s
v_M2_	15.0 μM/s
v_M3_	40.0 s^−1^
v_p_	0.05 μM/s
k_2_	0.1 μM
k_CaA_	0.15 μM
k_CaI_	0.15 μM
k_ip3_	0.1 μM
k_p_	0.3 μM
k_deg_	0.08 s^−1^
k_out_	0.5 s^−1^
k_f_	0.5 s^−1^
n	2.02
m	2.2

The ability of spontaneous oscillations to modulate synaptic activity can be demonstrated by applying the relationships established in Eqs. (7) and (8) to the LH model^26^. These equations, which describe the release of glutamate (*G*_m_) and ATP (*G*_a_) from astrocytes into the tripartite synapse, were obtained from the Postnov et al. model^10^ and are Ca^2+^ oscillations dependent. Glutamate depolarizes the postsynaptic neuron, whereas ATP hyperpolarizes it via adenosine. Table 2 lists the parameter values for Eqs. (7) and (8), obtained from the Postnov et al. work^10^.

7 $$\begin{aligned}{} & {} \tau _{Gm}\frac{dG_m}{dt} =[1+tanh(S_{Gm}(X-h_{Gm}))]\times (1-G_m)-\frac{G_m}{d_{Gm}} \end{aligned}$$

8 $$\begin{aligned}{} & {} \tau _{Ga}\frac{dG_a}{dt} =[1+tanh(S_{Ga}(X-h_{Ga}))]\times (1-G_a)-\frac{G_a}{d_{Ga}} \end{aligned}$$

**Table 2:** Parameter’s values for glutamate (7) and ATP (8) equations^10^.

Parameter	Value
$\tau$_Gm_	5.0
S_Gm_	100
h_Gm_	0.5
d_Gm_	3.0
$\tau$_Ga_	3.0
S_Ga_	100
h_Ga_	0.5
d_Ga_	3.0

**Figure 2:**
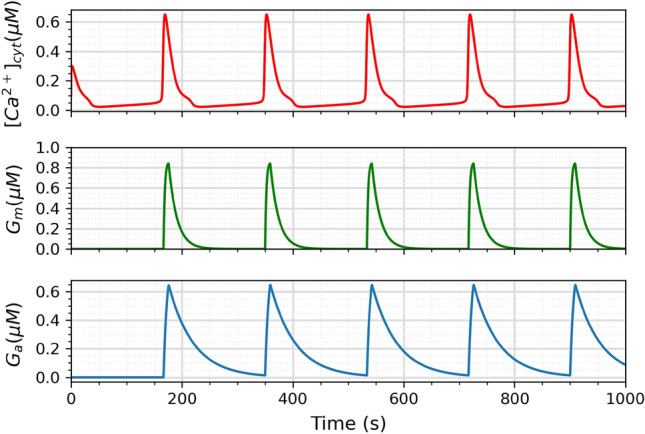
Release flux of glutamate and ATP as modulated by spontaneous Ca^2+^ oscillations.

The parameters in Table 2 are briefly explained below, and the flow of glutamate and ATP release by the spontaneous oscillations is presented in Fig. 2:

- $\tau _{Gm}$—controls the time scale for the glutamate process;
- *S*_Gm_—steepness of activation for the *G*_m_ function;
- *h*_Gm_—threshold parameter for the activation of glutamate release;
- *d*_Gm_—controls the deactivation rate of glutamate release;
- $\tau _{Ga}$—controls the time scale for the ATP process;
- *S*_Ga_—steepness of activation for the *G*_a_ function;
- *h*_Ga_—threshold parameter for the activation of ATP release;
- *d*_Ga_—controls the deactivation rate of ATP release.

**Figure 3:**
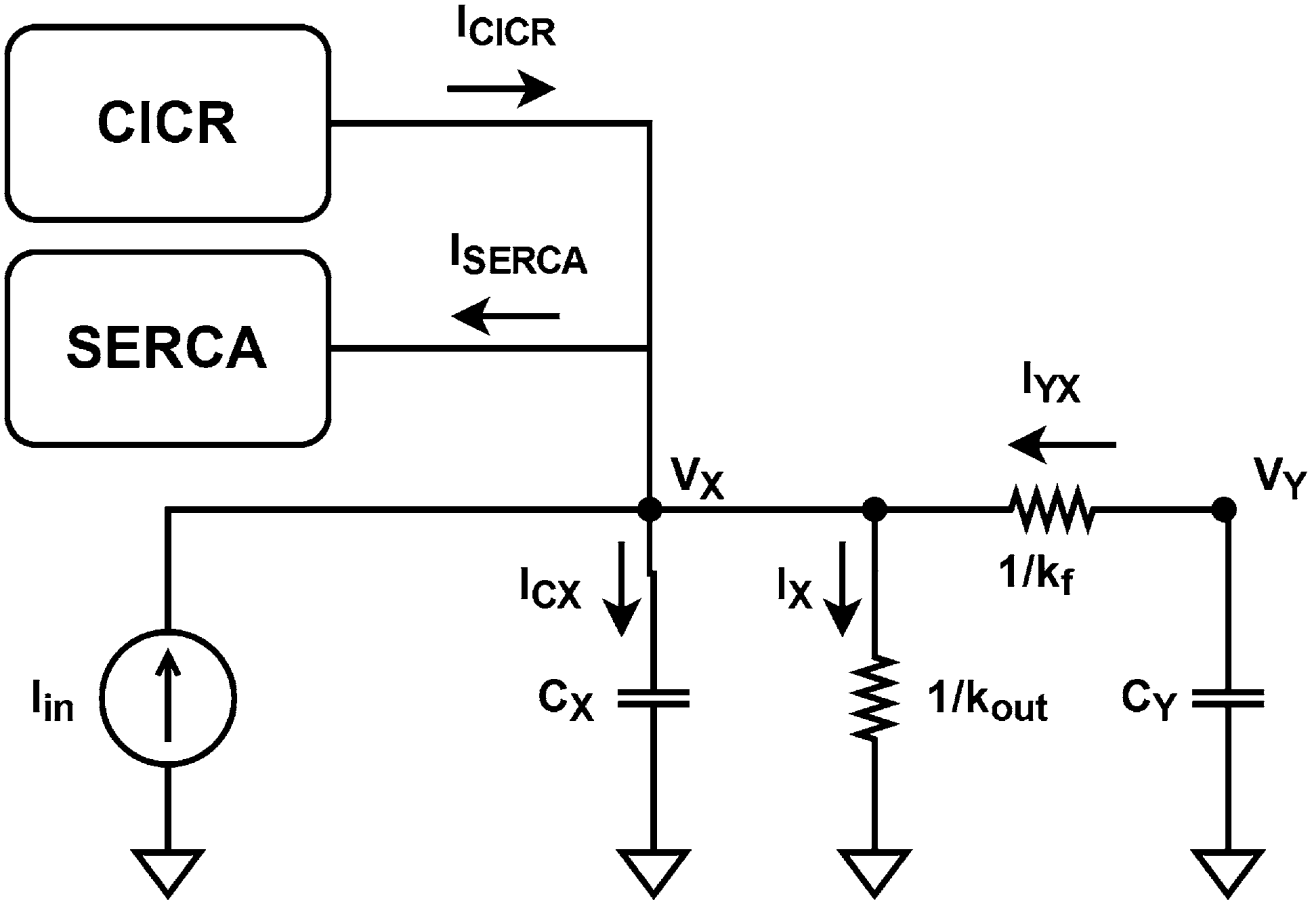
Proposed block diagram for [Ca^2+^]_cyt_, *V*_X_.

**Figure 4:**
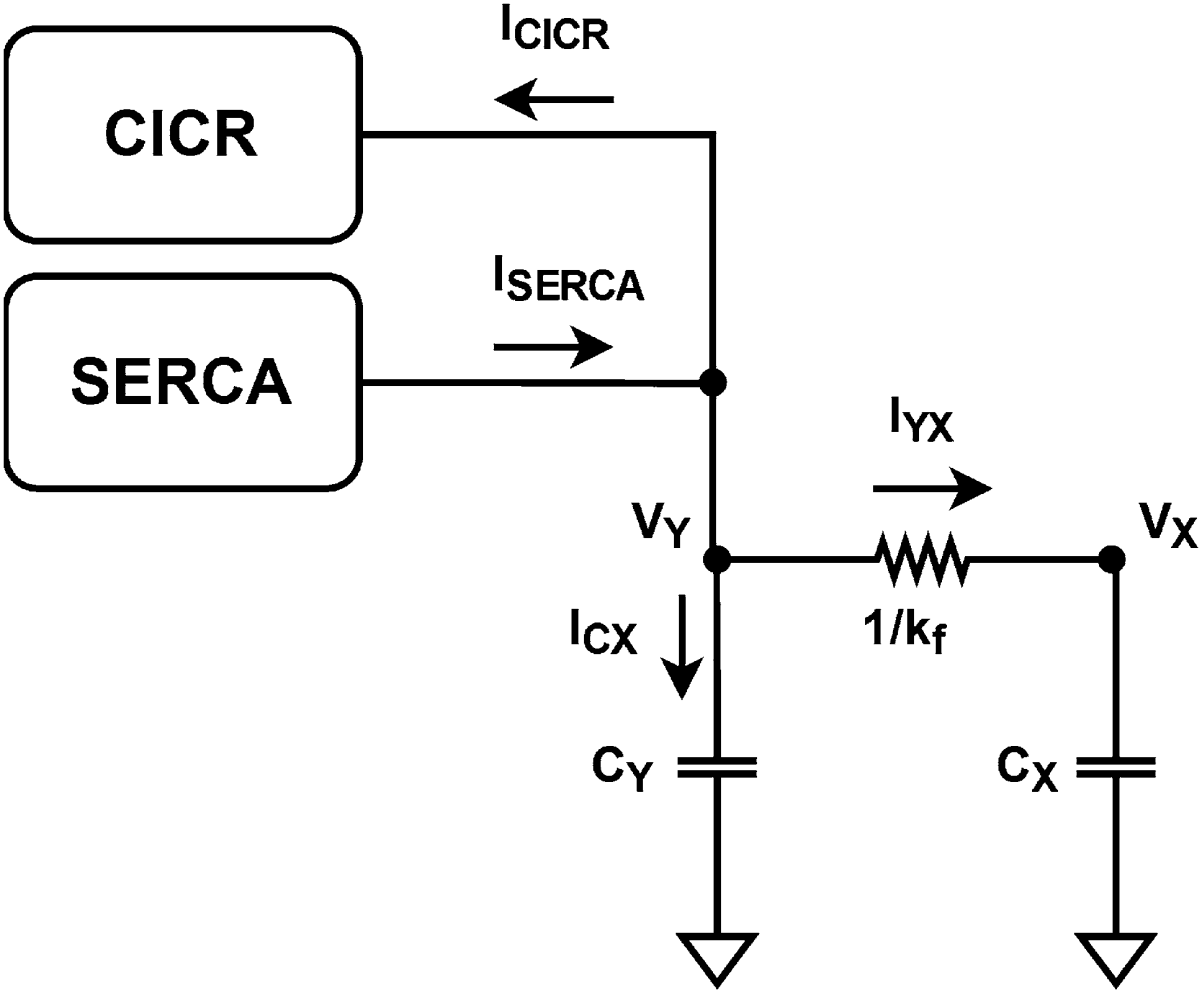
Proposed block diagram for [Ca^2+^]_ER_, *V*_Y_.

### Proposed circuit

This section details the design of an analog circuit that implements the spontaneous oscillations model. To convert a mathematical model into an analog circuit, the first step is to establish a correspondence between biological and electronic variables. Subsequently, fundamental circuit principles can be used to analyze the relationships between these variables.

LH’s mathematical model is a detailed codification of the behavior of spontaneous oscillations and the variables involved in their generation. This biological to electronic variable mapping is quite interesting for electronic circuit implementations, in which it is necessary to know the influence of each variable on the desired result. Therefore, as an initial approach, the implementation based on the mathematical model was adopted, observing the effect of each variable and the device and/or physical quantity that could physically approximate it. In further work still based on the LR model, some adjustments can be made to the circuit architecture in order to achieve oscillations closer to those provided by the mathematical equation. The idea is to minimize discrepancies as much as possible. For example, the linear part of the hyperbolic tangent can be optimized, some subcircuits that act as filters can be added to improve circuit performance, or even more precise current source subcircuits can be used. Additionally, a design strategy for spontaneous oscillation circuits can be formulated according to the interaction to be performed.

Subsection “General structure of the circuit” defines the corresponding representation between biological and electrical variables, and Eqs. (1–3) are rewritten accordingly. Kirchhoff’s current law (KCL) is then applied to the new equations to obtain the general structure of the model’s circuit.

The design of the circuits for the nonlinear terms SERCA, PLC, and CICR is discussed in Subsection “SERCA, PLC, and CICR modules”. These circuits are constructed by approximating the sigmoidal nonlinear terms with the hyperbolic tangent function, which can be implemented using a differential pair, a building block of analog electronics.

**Figure 5:**
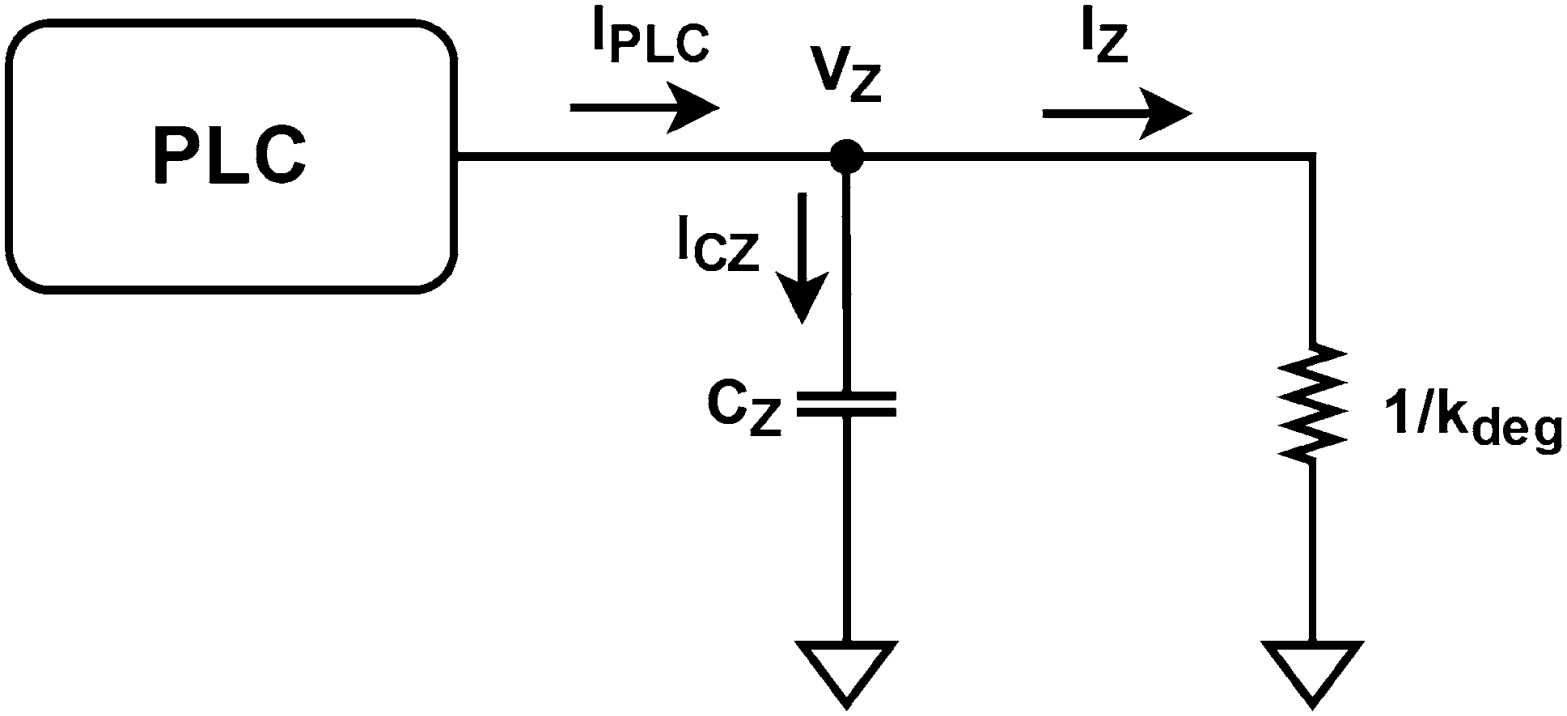
Proposed block diagram for [IP_3_]_cyt_, *V*_Z_.

### General structure of the circuit

The design of a neuromorphic circuit should take into consideration the device’s inherent physics capabilities and fundamental circuit laws. In an initial analysis, we observe that Eqs. (1) to (3) are composed of the sum of various terms. Kirchhoff’s laws state that the algebraic sum of either voltage or current is zero. Additionally, the current of a capacitor is given by the derivative of the voltage with respect to time, leading to the natural application of Kirchhoff’s current law (KCL) to the model. Thus, we introduce the following representation to rewrite the mathematical model as Eqs. (9) to (11):

1. Elements that represent concentration of ions or molecules are considered as differences in potential (voltage).
2. Elements that represent flux of ions or molecules are considered as current.

**Figure 6:**
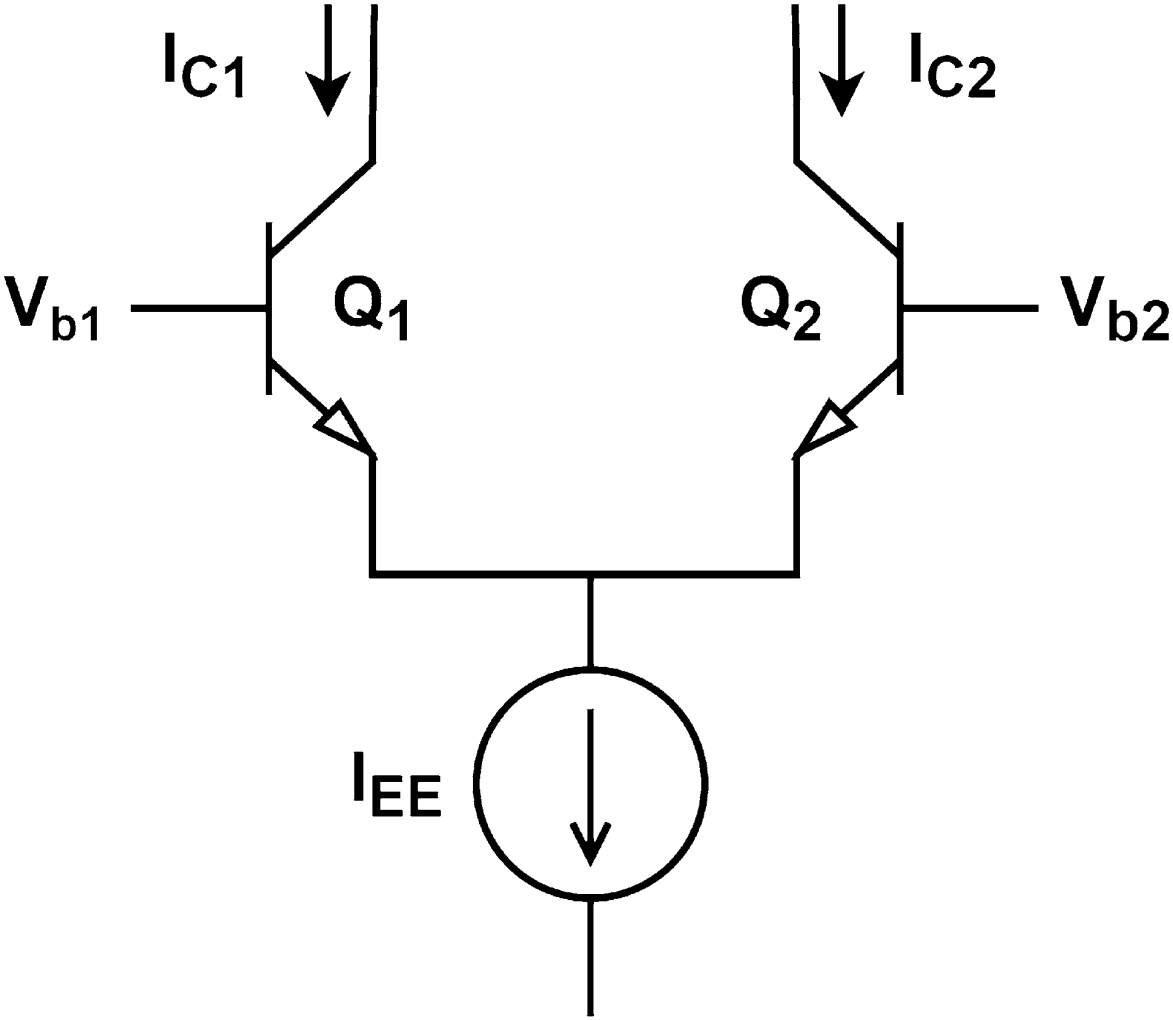
Bipolar differential pair schematic.

9 $$\begin{aligned}{} & {} c_X\frac{dV_X}{dt} =I_{in}-I_X+I_{CICR}-I_{SERCA}+I_{YX} \end{aligned}$$

10 $$\begin{aligned}{} & {} c_Y\frac{dV_Y}{dt} =I_{SERCA}-I_{CICR}-I_{YX} \end{aligned}$$

11 $$\begin{aligned}{} & {} c_Z\frac{dV_Z}{dt} =I_{PLC}-I_Z \end{aligned}$$

*V*_X_, *V*_Y_, and *V*_Z_ represent the primary variables *X*, *Y*, and *Z* as voltages, while their multiplying parameters *k*_out_, *k*_f_, and *k*_deg_ are transformed into conductances. The terms *V*_CICR_, *V*_SERCA_, and *V*_PLC_ are relabeled as *I*_CICR_, *I*_SERCA_, and *I*_PLC_, respectively, to indicate that the solutions to Eqs. (4) to (6) are viewed as output currents by the system. Because of their nonlinear nature, the circuits implementing these equations are designed as separate modules in the subsequent subsection.

To define capacitance, we introduce capacitor elements *c*_X_, *c*_Y_, and *c*_Z_ in the equations. Their values are assumed to be unit, thereby preserving the original equations. Finally, applying KCL to Eqs. (9), (10), and (11) produces the block diagrams presented in Figs. 3, 4, and 5, respectively.

### SERCA, PLC, and CICR modules

The SERCA, PLC, and CICR modules implement the nonlinear functions given by Eqs. (4), (5), and (6), which consist of Hill equations and are sigmoidal in nature. The Hill equation can be approximated by the hyperbolic tangent function, which can be easily implemented using a transistor differential pair.

The aim of this study is to present a preliminary investigation into the feasibility of the electronic implementation of spontaneous oscillations using simple devices and architectures. Building upon the previous representations stated as 1 and 2, the output signals of the proposed circuits are currents. Hence, for the initial approach, bipolar junction transistors (BJTs) were chosen as they are well-suited for low-current-driven circuits^36,37^. BJTs are current-controlled devices where the collector-emitter current is regulated by the base-emitter current.

The circuit design phase was initiated by selecting the parameters to be adjusted. It was decided to choose the parameters from the large-signal model of the BJT to construct a simple Spice model based on these values. The selected parameters were Is (saturation current), V$_{AF}$ (forward Early voltage), and $\beta _F$ (forward current gain). Initially, simulations were performed with typical values for these parameters. Subsequently, some empirical adjustments were made until the circuit provided oscillations that were as close as possible to the LH model. The parameters’ final values are in Tables 3 and 4. More parameters, like physical and geometrical ones, will be included in future work to adjust the circuit operation and make it closer to the reality of physical implementation. These parameters will encompass non-idealities inherent to transistor operation. Additionally, a parameter tuning technique for circuit optimization will be proposed. Figure 6 shows a schematic diagram of the differential pair based on bipolar junction transistors (BJTs). The differential current output is given by Eq. (12), where:

**Figure 7:**
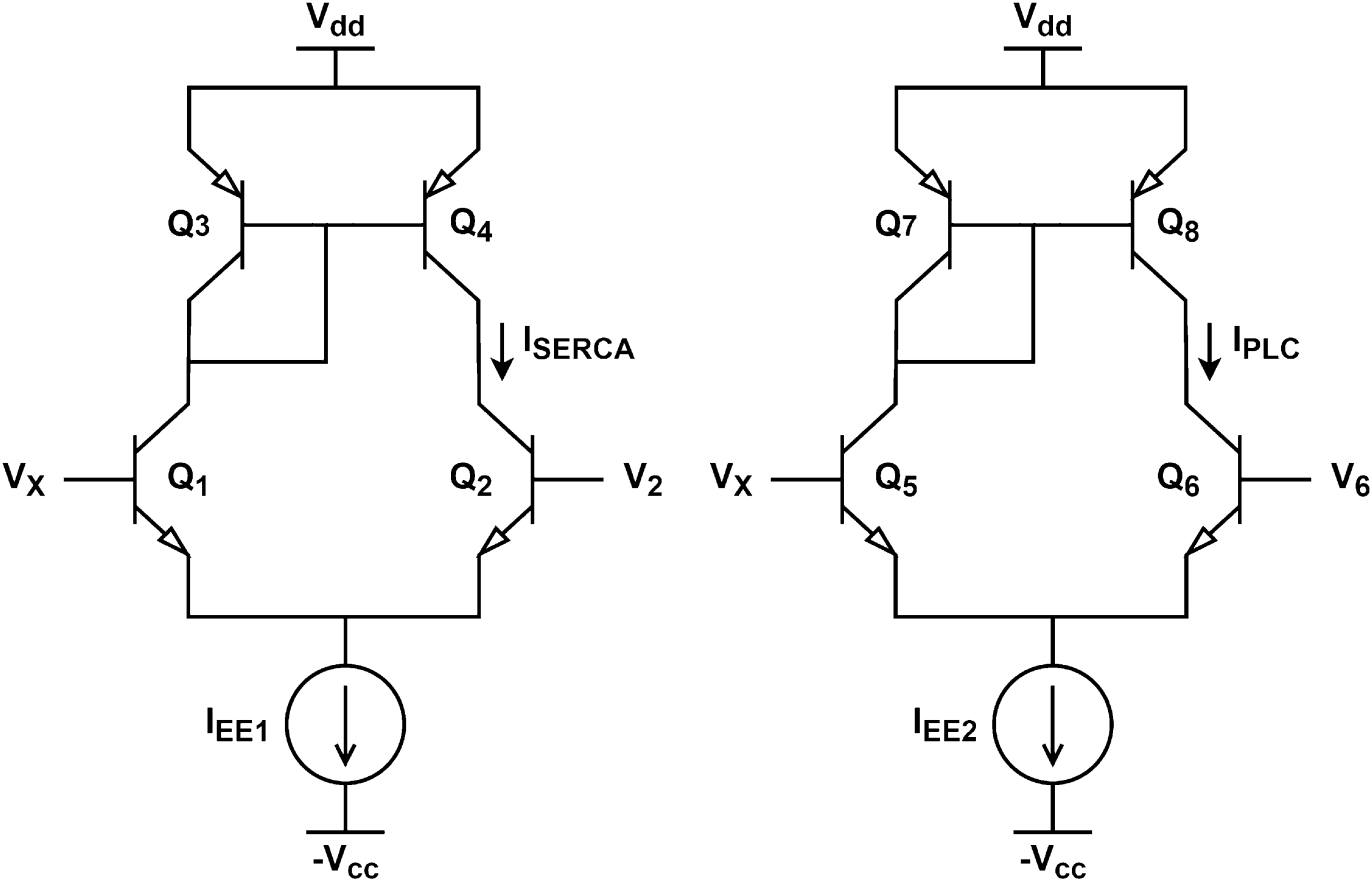
Differential pair schematics for (**left panel**) SERCA and (**right panel**) PLC modules.

- *Ic*_1_, *Ic*_2_—collector current of transistors Q1 and Q2, respectively;
- $\alpha$—common base current gain;
- *I*_EE_—current source;
- *V*_b1_, V_b2_—base voltage of transistors Q1 and Q2, respectively;
- *V*_T_—thermal voltage.

12 $$\begin{aligned} I_{c1}-I_{c2}=\alpha \, I_{EE} \, tanh\left( \frac{V_{b1}-V_{b2}}{2V_T}\right) \end{aligned}$$

Both SERCA and PLC equations are functions of one Hill equation each, thus presenting a similar module design. Using Eq. (12) to approximate Eqs. (4) and (5), we obtain Eqs. (13) and (14), respectively. These equations are used to design the differential pairs with active load modules depicted in Fig. 7. The following BJT parameters were adjusted: saturation current, forward Early voltage, and forward beta. The proposed circuit parameters are presented in Table 3.

13 $$\begin{aligned}{} & {} I_{SERCA}\approx tanh(5X-0.065) \end{aligned}$$

14 $$\begin{aligned}{} & {} I_{PLC}\approx tanh(1.78X-0.045) \end{aligned}$$

**Table 3:** Parameter’s values for SERCA and PLC circuits.

Parameter	Value
I_S_	$5\times 10^{-15}$ A
(V_af_)_Q1, Q2, Q3, Q4_	200 V
(V_af_)_Q5, Q6, Q7, Q8_	100 V
(b_f_)_Q1, Q2, Q5, Q6, Q7, Q8_	300
(b_f_)_Q3, Q4_	100

The CICR module presents a more complex equation than the SERCA and PLC modules, as it contains a rational function and two Hill equations. To design the CICR module, its function was decomposed into three separate equations. The equations dependent on *X* are called *F*_X1_ (16) and *F*_X2_ (17), and the equation dependent on *Z* is named *F*_Z_ (18). Also, the term $(Y-X)$ from the original Eq. (6) is replaced by $\frac{I_{YX}}{k_f}$.

15 $$\begin{aligned}{} & {} I_{CICR}=4v_{M3}\,F_{X1}\, F_{X2}\,F_Z\,\frac{I_{YX}}{k_f} \end{aligned}$$

16 $$\begin{aligned}{} & {} F_{X1}=\frac{k_{CaA}^n}{X^n+k_{CaA}^n} \end{aligned}$$

17 $$\begin{aligned}{} & {} F_{X2}=\frac{X^n}{X^n+k_{CaI}^n} \end{aligned}$$

18 $$\begin{aligned}{} & {} F_Z=\frac{Z^m}{Z^m+k_{ip3}^m} \end{aligned}$$

**Figure 8:**
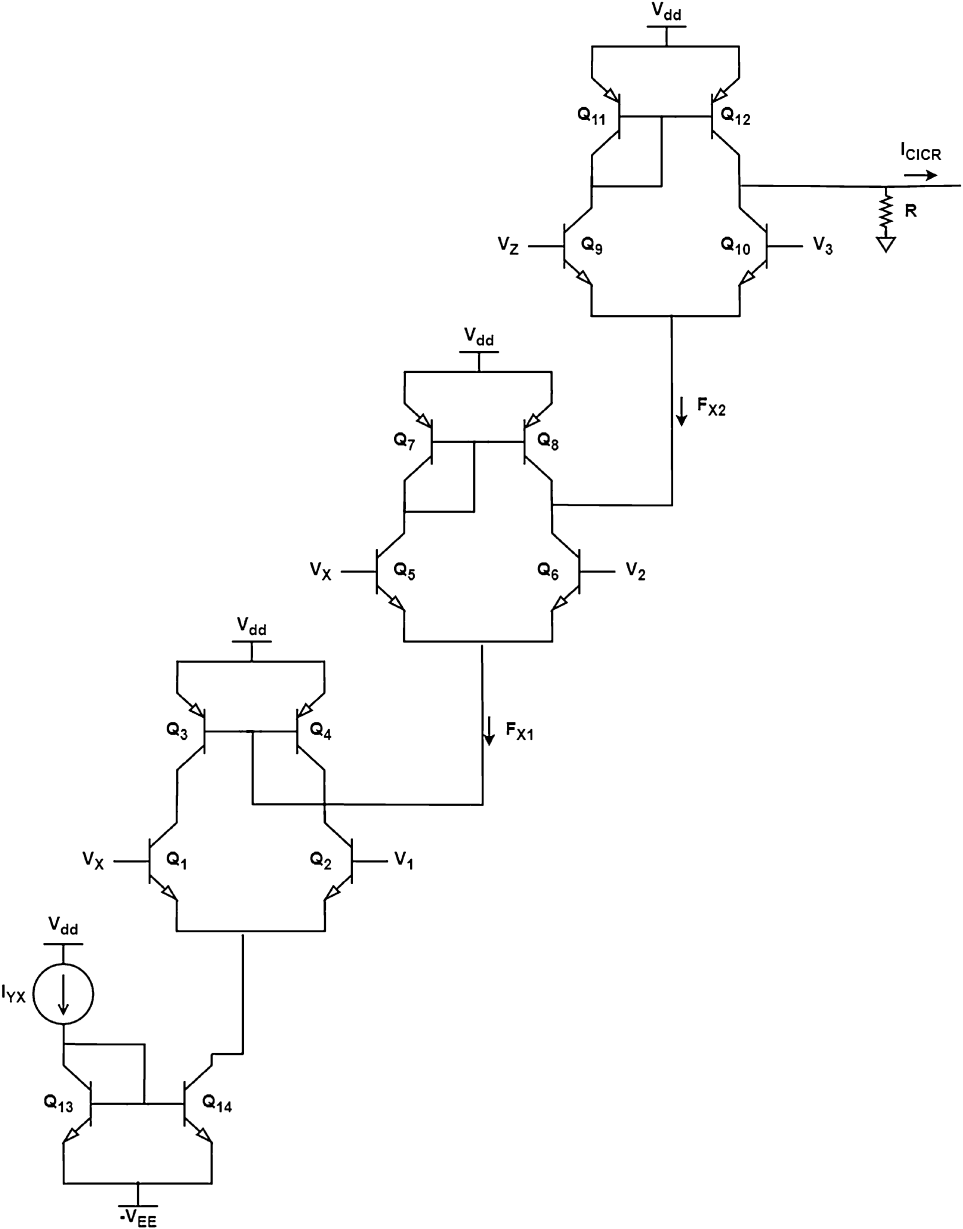
Circuit schematic for CICR module.

The equation *F*_X1_ can be approximated by the negative hyperbolic tangent (Eq. 20), which is an odd function that can be expressed as Eq. (19) by simply taking its negative. We approximate *F*_X1_ using Eq. (20), and the current addition of 1.08 in Eq. (20) can be implemented using an independent current source. In contrast, *F*_X2_ is a regular Hill equation and can be implemented without alterations to the differential pair circuit. The hyperbolic tangent approximation equation for *F*_X2_ is shown in Eq. (21).

19 $$\begin{aligned}{} & {} I_{c2}-I_{c1}=\alpha \, I \, tanh\left( \frac{V_{B2}-V_{B1}}{2V_T}\right) \end{aligned}$$

20 $$\begin{aligned}{} & {} F_{X1}\approx tanh(0.22-5.5X)+1.08 \end{aligned}$$

21 $$\begin{aligned}{} & {} F_{X2}\approx 4\,tanh(3.9X-0.07) \end{aligned}$$

Similarly, *F*_Z_ (as given by Eq. 18) is also a regular Hill equation and can be approximated using the hyperbolic tangent function, as shown in Eq. (22).

22 $$\begin{aligned} F_Z\approx 5\,tanh(2.5Z-0.03) \end{aligned}$$

**Figure 9:**
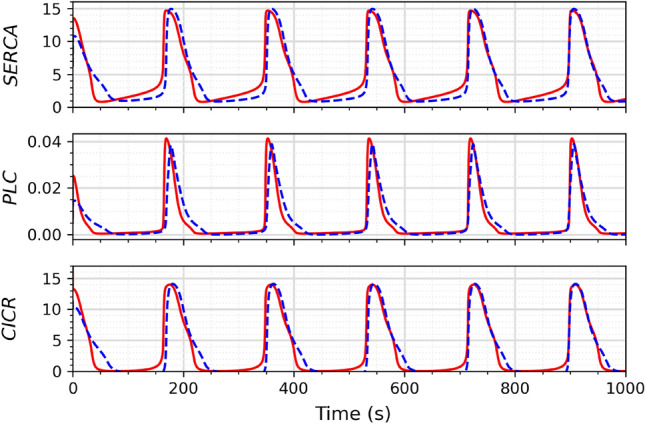
Output from SERCA, PLC, and CICR modules. The original model is shown in solid red lines and circuit simulation result in dotted blue lines.

**Figure 10:**
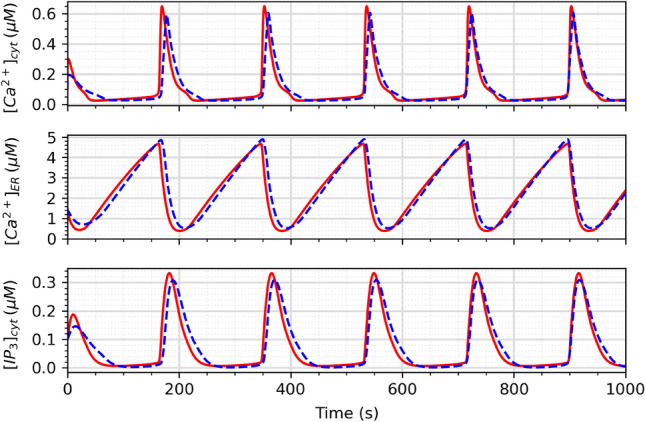
Graph showing [Ca^2+^]_cyt_, [Ca^2+^]_ER_, and [IP_3_]_cyt_ variables for the mathematical model (red line) and the circuit simulation (blue dotted line).

**Figure 11:**
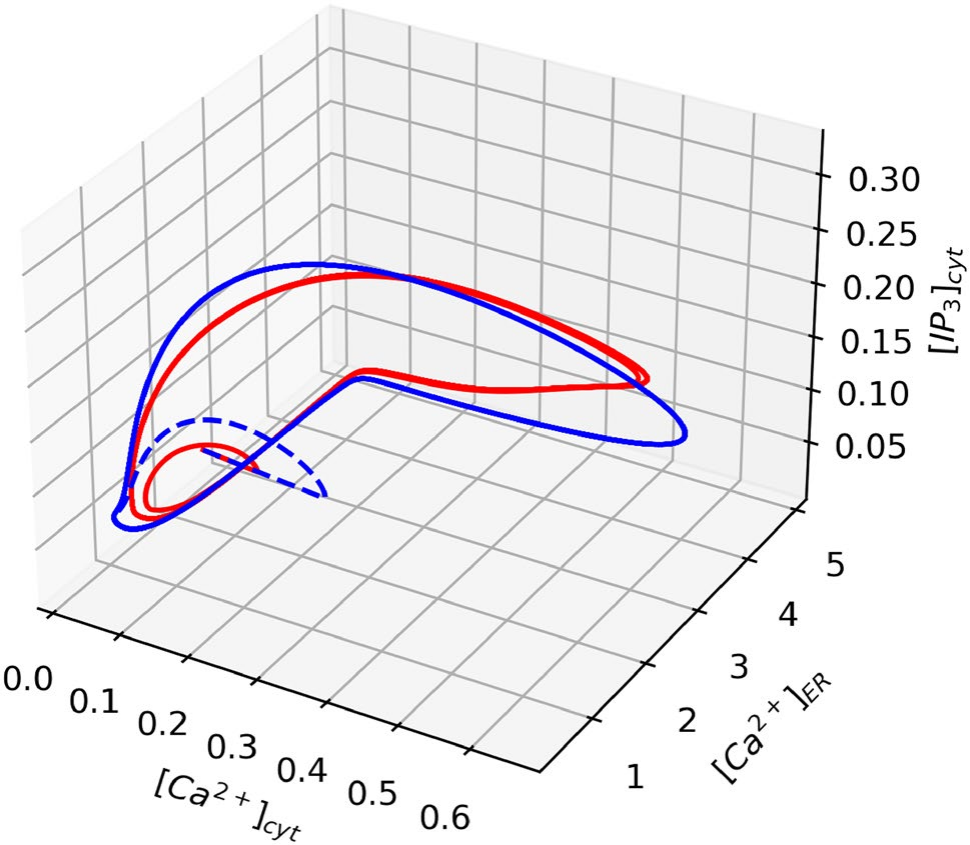
Phase space diagram comparison of mathematical model (red line) and the circuit (blue line).

Finally the *I*_CICR_ complete module’s circuit is shown in Fig. 8. The full circuit includes the differential pairs for the three nonlinear functions and a current mirror (transistors Q_13_ and Q_14_) that inputs $I_{YX}$ as the differential pair current source. The resistance R is added at the circuit output to supply the term $4\,v_{M3}\,\frac{1}{k_f}$, making the equation complete. The circuit parameters are given in Table 4.

**Table 4:** Parameter’s values for CICR circuit.

Parameter	Value
R	$12.5 \times 10^{-3} \Omega$
I_S_	$5\times 10^{-15}$ A
(V_af_)_Q1, Q2, Q3, Q4, Q13, Q14_	350 V
(V_af_)_Q5, Q6, Q7, Q8, Q9, Q10, Q11, Q12_	100 V
(b_f_)_Q1, Q2, Q3, Q4, Q13, Q14_	300
(b_f_)_Q5, Q6, Q7, Q8, Q9, Q10, Q11, Q12_	100

## Results

The circuits depicted in Figs. 7 and 8 were simulated using the LTspice simulator with a bias voltage of $\pm 1.5$ V. The simulation results were compared with the dynamics of the spontaneous Ca^2+^ oscillations model (Eqs. 1–6), which were simulated in Python using NumPy^38^ and Matplotlib^39^. The simulations of the circuits shown in Figs. 7 and 8 are presented in Fig. 9 and are compared with the mathematical model. The proposed circuit shows a good output match with the original mathematical model.

Figure 10 shows the time evolution of the variables [Ca^2+^]_cyt_, [Ca^2+^]_ER_, and [IP_3_]_cyt_ from both the circuit and mathematical models. The Ca^2+^ oscillations obtained in the circuit closely match the theoretical model, demonstrating the high accuracy of the astrocyte circuit. Figure 11 presents the phase space portrait for both the model and circuit, which exhibit similar dynamics and stability, indicating that the circuit is a good representation of the biological model dynamics.

**Figure 12:**
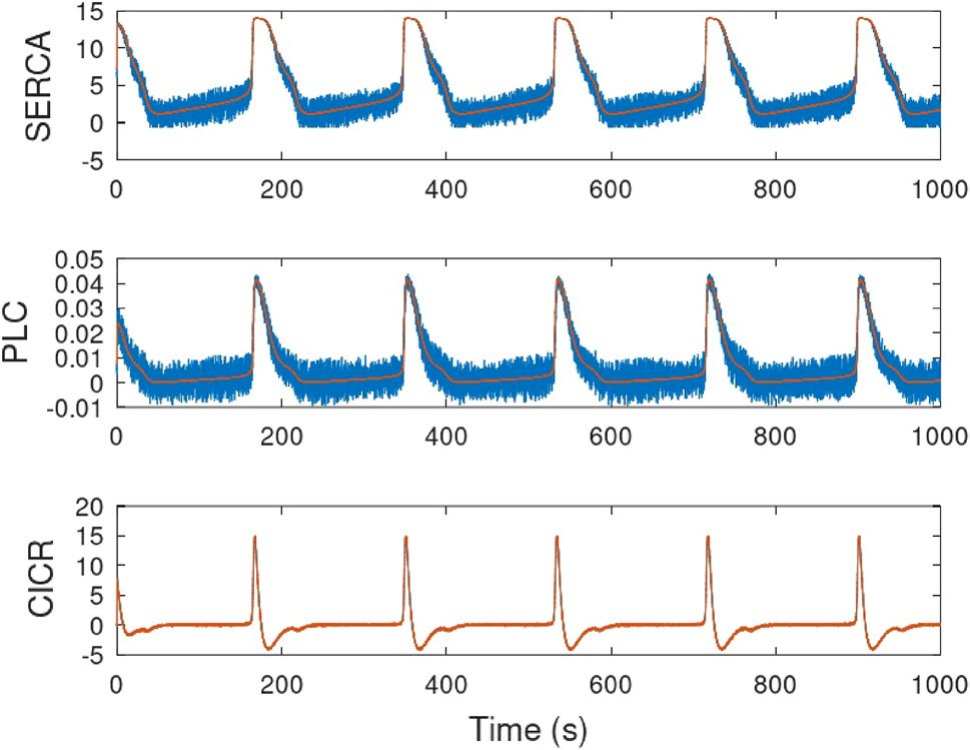
Output from SERCA, PLC, and CICR modules for ideal inouts and noisy inputs. The ideal outputs are shown in solid red lines and noisy result is in solid blue lines.

**Figure 13:**
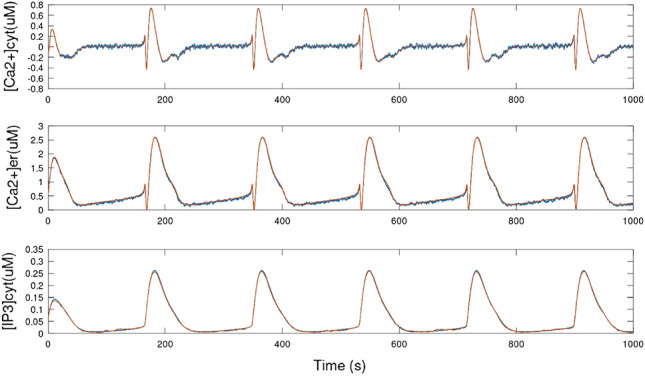
[Ca^2+^]_cyt_, [Ca^2+^]_ER_, and [IP_3_]_cyt_ variables for ideal inputs (red line) and noisy inputs (blue line).

**Figure 14:**
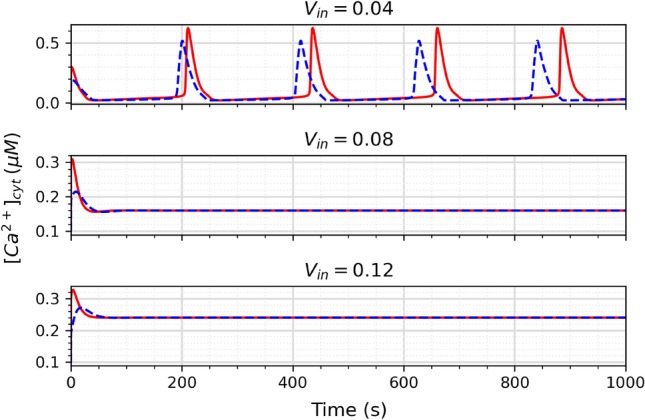
Ca^2+^ oscillations for different values of *V*_in_. The red solid line represents the mathematical model, and the blue dotted line represents the proposed circuit.

**Figure 15:**
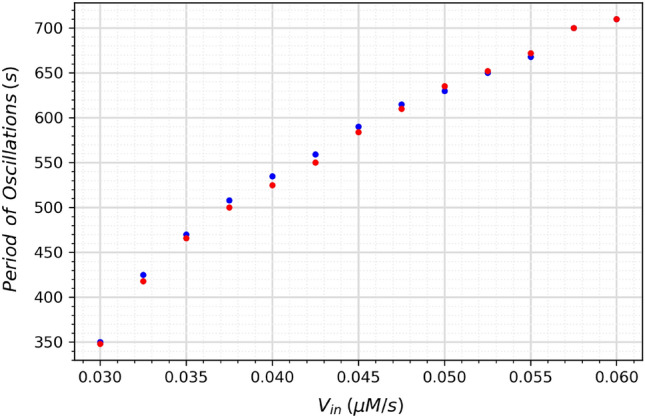
Period of oscillations as a function of V_in_ for the neuromorphic circuit. The red dots represent the mathematical model, and the blue dots represent the proposed circuit.

**Figure 16:**
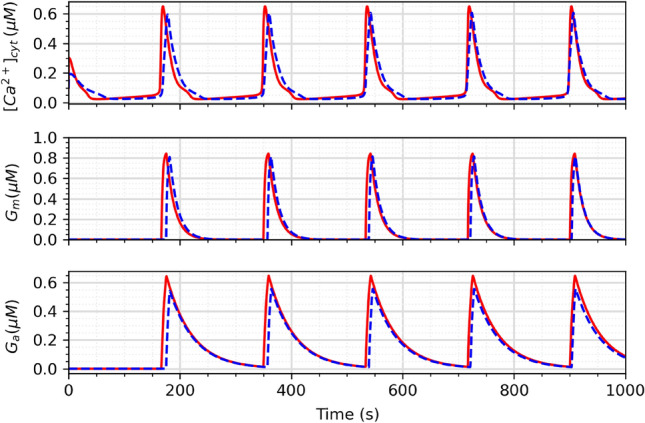
G_m_ and G_a_ release in the tripartite synapse due to Ca^2+^. The red solid line represents the mathematical model, and the blue dotted line represents the proposed circuit.

Random noise was added to the input signals of the proposed circuit to evaluate its robustness. The added noise did not exceed 10$\%$ of the maximum magnitude of the input signal. As can be seen in Fig. 12, the SERCA and PLC signals generated by the proposed circuit clearly exhibited the effects of the input noise. However, the CICR signal was hardly affected by it and remained very close to the ideal value. The circuit architecture of this subcircuit has several hyperbolic tangent modules, which likely absorbed the effects of small input variations due to noise.

On the other hand, [Ca^2+^]_cyt_, [Ca^2+^]_ER_, and [IP_3_]_cyt_ (Fig. 13)exhibited subtle effects from the presence of noise. This can also be explained by the filtering effect of the resistive and capacitive elements in the output modules of each oscillation.

The circuit was also simulated with different values of *V*_in_ to investigate the astrocyte’s ability to form spontaneous oscillations in different scenarios of extracellular calcium ion flow. Figure 14 shows the results for the values of 0.04, 0.08, and 0.12 for *V*_in_. The graph shows a discrepancy between the original model and the circuit, specially in relation to the frequency for *V*_in_. Figure 15 shows how the period of oscillations changes as a function of V_in_ for the neuromorphic circuit. Despite the tendency of slightly higher frequency for the circuit, these results generally agree with the findings of Parri et al.^30^ regarding the spontaneous oscillations dependency of V_in_. Because it is still not known how exactly the astrocytes signalling, specially the spontaneous ones, affects behaviour and cognition, it is hard to estimate what effect this discrepancy would have on a neural network. However, the circuit still presents itself as a good tool to further study these oscillations.

Despite neural communication in Spiking Neural Networks being carried out in the form of noise-robust spikes, the feedback network formed by astrocytes is expected to enhance the robustness of neuron-astrocyte circuits. Furthermore, design techniques aimed at reducing noise are being considered. These techniques include the use of low collector current values, high $\beta$ values, and the application of filters and other appropriate techniques as needed^40^.

As the primary focus of this study is to demonstrate the possibility of generating spontaneous oscillations using electronic circuits, a preliminary estimation of the dissipated power was conducted. The simulation results indicate that the dissipation is approximately 40 μW. The circuit proposed in this work exhibits power dissipation on the same order of magnitude as the values presented for neurotransmitter-evoked Ca2+ oscillation^24^. Future improvements in this value are expected by employing low-power transistors, such as CMOS or carbon nanotube transistors, instead of the chosen BJTs. It should be noted, however, that the desired modulation characteristics of astrocytes may require implementations with mixed technologies.

Finally, Fig. 16 compares the release of glutamate and ATP in the tripartite synapse between the mathematical model and the proposed circuit. The Ca^2+^ oscillations generated by the proposed circuit is able to trigger a release response of glutamate and ATP that is very similar to that of the original mathematical model, achieving the main goal of this study.

## Conclusion

In this work, we proposed and successfully implemented an analog neuromorphic circuit capable of spontaneous Ca^2+^ oscillations. This circuit exhibits similar dynamic behavior compared to the original biological model, even when subjected to noisy inputs. It is important to highlight that the proposed analog solution is simpler and easier to use in certain applications, as it does not require FPGA programming for implementing oscillations, unlike some previous digital circuit proposals. Another point to be highlighted is that the proposed architecture is capable for reproducing the similar dynamics of the biological model in reasonable accuracy.

The initial goal has been successfully achieved. As a first approach, bipolar transistor technology was chosen, but it is not limited to this device type. The SERCA, PLC, and CICR equations can be implemented with other devices that generate a hyperbolic tangent output, such as CMOS technology operating in the weak inversion zone. Some modules of the proposed circuit can be improved, and others can be added to enhance performance. The proposed circuit can be implemented in specific tasks to analyze self-repair properties, such as in robotics-related applications.

Moving forward, the objective is to further enhance the proposed implementation with a focus on large-scale integration. Parameters such as power consumption and robustness will be taken into account to optimize the proposed architecture. Additionally, a comprehensive methodology for designing spontaneous oscillation circuits can be proposed and used based on the required astrocyte-neuron interaction.

## Data Availability

The datasets utilized and/or examined throughout the present study are accessible upon reasonable request from the corresponding author.
